# The *763C>G* Polymorphism of The Secretory *PLA2IIa* Gene Is Associated with Endometriosis in Iranian Women

**DOI:** 10.22074/ijfs.2015.4184

**Published:** 2015-02-07

**Authors:** Mehdi Sahmani, Masoud Darabi, Maryam Darabi, Talaat Dabaghi, Safar Ali Alizadeh, Reza Najafipour

**Affiliations:** 1 Department of Clinical Biochemistry and Medical Genetics, Cellular and Molecular Research Center, Qazvin University of Medical Sciences, Qazvin, Iran; 2 Department of Biochemistry and Clinical Laboratories, Faculty of Medicine, Tabriz University of Medical Sciences, Tabriz, Iran; 3 Kosar Hospital, Qazvin, Iran

**Keywords:** Endometriosis, polymorphism genetic, *PLA2G2A*

## Abstract

**Background:**

Endometriosis is a chronic gynecological disease resulting from complex interactions between genetic, hormonal, environmental and oxidative stress and intrinsic inflammatory components. The aim of this study was to investigate the potential association of the *763C>G* polymorphism in the secretory phospholipase A2 group IIa gene (*PLA2G2A*) with the risk of endometriosis in Iranian women.

**Materials and Methods:**

Ninety seven patients with endometriosis along with 107 women who were negative for endometriosis after laparoscopy and laparatomy, and served as the control group, were enrolled for this cross-sectional study. Samples were genotyped using the polymerase chain reaction-restriction fragment length polymorphism method.

**Results:**

Multivariate analysis was used to examine the association between the risk of endometriosis and the *763C>G* polymorphism of *PLA2G2A*. Genotype distributions of *PLA2G2A* were significantly different between patients and the controls (p<0.001, OR=0.22, 95% CI=0.21-0.39). Correlation analysis showed that there was a significant association between the normal homozygous genotype and susceptibility to endometriosis (p<0.001).

**Conclusion:**

The present study suggests that the *763C>G* polymorphism of *PLA2G2A* plays an important role as an independent factor in the risk of endometriosis in Iranian women.

## Introduction

Endometriosis is one of the most frequent disorders in gynecology that is characterized by development of endometrial tissue outside the uterus (1). Endometriosis affects >10% of all women of reproductive age and >30% of all infertile women (2, 3). The common symptoms of endometriosis are pelvic pain, dysmenorrhoea, dyspareunia and infertility (4). The disease is diagnosed by laparoscopy with or without biopsy for histological diagnosis (4, 5). According to extent, endometriosis is classified as stage I (minimal), stage II (mild), stage III (moderate) and stage IV (severe) (6). Previous studies have demonstrated that oxidative stress and inflammatory activity as well as genetic abnormalities may be associated with the development and progression of endometriosis (2, 7, 8).

Similar to the process that exists in cardiovascular disease, an abnormal lipid profile including increased low-density lipoprotein (LDL) , decreased high density lipoprotein (HDL) and subsequently decreased HDL and, production of oxidized LDL (oxLDL) particles in peritoneal fluid ,could be involved in the development of endometriosis (9, 10). Melo et al showed that the abnormal lipid profile with elevated LDL and Non-HDL may increase oxidative stress and inflammation in the peritoneal fluid of women and subsequently the risk of endometriosis (11). Secretory phospholipase A2 group IIa (s*PLA2IIa*) is a superfamily of enzymes that hydrolyse the sn-2 ester bond of glycerophospholipids to produce non-esterified free fatty acids (NEFAs) and lysophospholipids (12).

Release of NEFA arachidonic acid (AA) is a key step as a precursor in the production of eicosanoids such as leukotrines, thromboxanes and prostaglandin E2 (13). This process therefore promotes the production of pro-inflammatory lipid mediators which aid the initiation and maintenance of prolonged inflammatory responses in the body (14, 15). Also studies have shown that overexpression of human s*PLA2IIa* results in increased lesion size and oxidative stress (16, 17). The human s*PLA2IIa* gene (*PLA2G2A*) is located on chromosome 1p34-36. Several single nucleotide polymorphisms (SNP) previously identified, capture 92% of the variation of *PLA2G2A* (18).

The *763C>G* polymorphism (rs11573156), in the 5´UTR which lies in exon 2 of *PLA2G2A* , showed strong association with s*PLA2IIa* levels and coronary artery disease (CAD) risk (19-21). Several studies have found association between s*PLA2IIa* gene polymorphisms and CAD risk (18, 22, 23) but no study has been conducted on endometriosis. The aim of this study was to determine the prevalence of the *763C>G* polymorphism of *PLA2G2A* in women with endometriosis with respect to the control group and its relationship with the risk of endometriosis in the Iranian population.

## Materials and Methods

### Subjects

Women with chronic pelvic pain or infertility referred to the Kosar Hospital in Qazvin, Iran for diagnostic laparoscopy between April 2011 and April 2012 were recruited for this cross-sectional study. Among 310 women, 204 were eligible and consented to participate in the study. Endometriosis patients and the control group were aged between 18 and 42. To obtain a more homogeneous population and verify that no endometriosis is present, controls were also selected from women undergoing laparoscopy. A total of 97 patients had surgical and histological evidence of endometriosis, while 107 patients without the disease served as controls (women with uterine myoma, dermoid cyst, paraovarian cyst, serous cyst and healthy women). Endometriosis was confirmed by diagnostic laparoscopy or laparotomy in both groups. In the endometriosis group, stage of the disease was diagnosed according to the revised American Fertility Society Classification (6).

Among the endometriosis patients, patients were diagnosed with stage I, 13 patients with stage II, 35 patients with stage III 10 and 39 patients with stage IV. None of the patients had received hormone therapy during the previous year. Women who had received anti-inflammatory drugs and contraceptives in the past three months, or had urological disease, endocrine disorders, familial dyslipidemia and chronic inflammatory were not included in the study. The study was approved by the ethics committee of Qazvin University of Medical Sciences.

### Assay of plasma lipid

Plasma total cholesterol (TC), High density lipoprotein-cholesterol (HDL-C) and triglyceride (TG) were assed using enzymatic method. (Selectra XL-VITA lab. Holland). Low-density lipoprotein- cholesterol (LDL-C) was calculated using the Friedewald equation: (LDL-C=total cholesterol (TC) - (HDLC) + TG/5) (24). All samples were stored at -70˚C for later simultaneous measurements.

### Genomic DNA analysis

Genomic DNA was extracted from the leukocytes of blood samples using the Qiagen DNA purification kit (Qiagen, USA). An 86 bp sequence of the s*PLA2IIa* gene was amplified by polymerase chain reaction (PCR) in a DNA thermal cycler (ABI, Veriti, USA) using oligonucleotide primers F: 5´- CAGCCTTGTGCCTCACCTA -3´ and R: 5´ CAGGCCGTCTTGTTTGTTCTG -3´.

**Fig 1:**
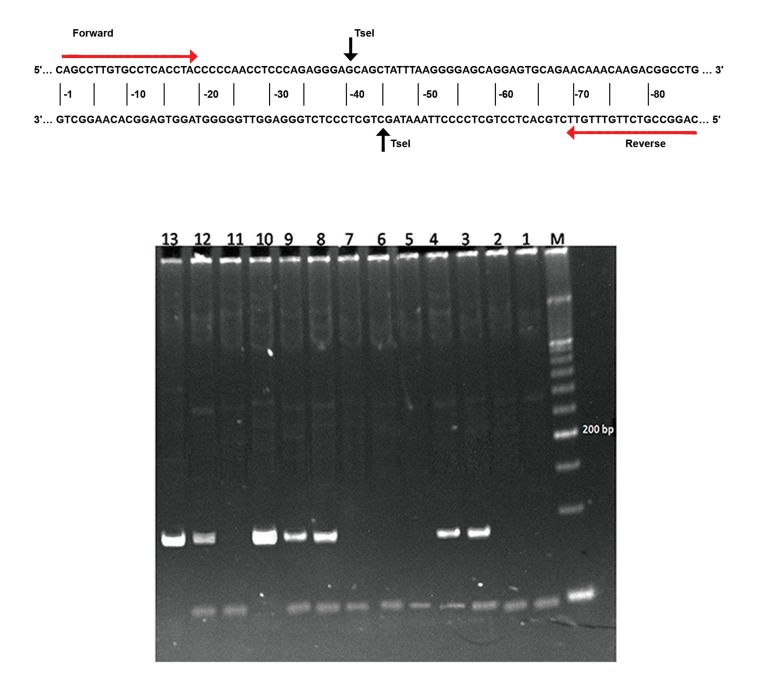
The ischemic picture s*PLA2IIa* gene of the amplicon, the position of the *763C>G* polymorphism, the place of the restriction enzyme with its recognition site as well as the location of primers. Gel picture analysis with the restriction enzyme TseI of *PLA2G2A* *763C>G* genotypes in genomic DNA of the study subjects. Lane M: molecular weight marker 50 bp; lanes 10 and 13:homozygous GG; lanes 3, 4, 8, 9, 12: heterozygous CG: lanes 1, 2, 5, 6, 7, 11: homozygous wild-type CC.

The PCR conditions were 94˚C for 5 minutes, 35˚C cycles of 94˚C for 45 seconds, 55˚C for 1 minute,72˚C for 1 minute, followed by 72˚C for 7 minutes (18). TseI enzyme (New England Biolabs, Inc., Beverly, MA) was used as a restriction enzyme. Samples were electrophoresed in polyacrylamide gel and the gel was then visualized by standard staining method (Fig 1). Expected fragment size combinations were 86 bp band (rare homozygotes), 86/46/40 bp (heterozygotes) and 46/40 bp (common homozygotes).

### Statistical analysis

Values were presented as mean ± SD, and statistical significance was defined as p values less than 0.05 (p≤0.05). Statistically significant differences in mean between genotypes were assessed by t test. Multivariate analysis was used to compare variable parameters between groups. Logistic regression analyses were performed for evaluating genotype distribution with respect to the presence of endometriosis as a dependent variable. All analyses were carried out using the statistical package for social sciences version 11.0 (SPSS, Chicago, IL, USA).

## Results

Demographic and metabolic parameters of patients and controls are shown in table 1. The mean of age for the endometriosis and control groups were 29.8 ± 5.4 and 29.5 ± 5.5 years respectively, (p=0.66). The body mass index (BMI) of the endometriosis group (25.1 ± 3.3 kg/m^2^) was significantly different from the control group (26.9 ± 3.9 kg/m^2^) (p=0.001), while for, waist circumference there was no such difference (endometriosis: 80.6 ± 9.1 cm vs. control: 81.2 ± 9.7 cm, p=0.69). The total cholesterol (TC) and LDL levels of the endometriosis group was significantly higher than the control group (p<0.001). Similarly, HDL levels were higher in the endometriosis group (46±10 mg/dL vs. 40 ± 9 mg/dL, p<0.001). TG levels were higher in the endometriosis group but was not significantly different between the two groups (128 ± 48 mg/dL vs. 127 ± 47 mg/dL, p=0.86).The genotype distributions of both groups were in Hardy-Weinberg equilibrium (p>0.05). Chi-square analysis between genetic groups identified that normal genotypes have more susceptibility than those carrying rare alleles (p<0.001).

The distribution of genotypes was different between the endometriosis group and the control group (68.4% CC, 29.5% CG, 2.1% GG vs. 31.8%, 51.4%, 16.8% respectively, p<0.001) (Table 2). By logistic regression analysis, the risk of endometriosis in different genetic groups was calculated (Table 3). The analysis showed that in the normal allele homozygous genotype, the risk of endometriosis was more than the other groups. Moreover, the analysis, after adjustment for factors such as BMI, HDL-C and LDL-C, the risk of endometriosis in patients with normal homozygous genotype was more than in those with the rare allele (p<0.001,OR=0.30,95% CI=0.14-0.63). Analysis of variance showed that in women with endometriosis, there is an increase in total TC and LDL-C compared with the control group (p<0.001, Table 4).

**Table 1:** Demographic and metabolic parameters of patients with endometriosis and the control group


	Control (n=107)	Endometriosis (n=95)	P

**Age (Y)**	29.5 ± 5.5	29.8 ± 5.4	0.66
**Body mass index (kg/m^2^)**	26.9 ± 3.9	25.1 ± 3.3	0.001
**Waist (cm)**	81.2 ± 9.7	80.6 ± 9.1	0.69
**Cholesterol (mg/dl)**	175 ± 30	216 ± 38	<0.001
**Triglyceride (mg/dl)**	127 ± 47	128 ± 48	0.86
**HDL-C (mg/dl)**	40 ± 9	46 ± 10	<0.001
**LDL-C (mg/dl)**	101 ± 20	130 ± 22	<0.001


Values are mean ± standard deviation. HDL-C; High density lipoprotein-cholesterol and LDL-C; Low-density lipoprotein-cholesterol.

**Table 2:** Genotype distributions in patients with endometriosis versus control


Gene	Control (n=107)	Endometriosis (n=95)	P

**PLA2G2A (%)**		
**CC**	34 (31.8%)	65 (68.4%)	
**CG**	55 (51.4%)	28 (29.5%)	
**GG**	18 (16.8%)	2 (2.1%)	


P value: Chi-square test. CC; Common homozygotes, CG; Heterozygotes and GG; Rare homozygotes.

**Table 3:** Logistic regression analysis of individual alleles with respect to the presence of endometriosis


	Univariate			Multivariate*		
	OR	95% CI	P	OR	95% CI	P

**PLA2G2A**	0.22	0.12-0.39	<0.001	0.30	0.14-0.63	0.001


Values are 95% confidence intervals (95%CI) of the odds ratios (OR). *Adjusted for body mass index (BMI), HDL-C and LDL-C.

**Table 4:** Metabolic parameters according to genotypes


	PLA2G2A			
	CC	CG	GG	P

**N**	99	83	20	
**Age (Y)**	29.8 ± 5.5	29.3 ± 4.6	30.7 ± 5.5	0.55
**Body mass index( kg/m^2^)**	25.5 ± 3.2	26.5 ± 4.0	26.4 ± 4.8	0.19
**Waist (cm)**	81.9 ± 8.5	80.5 ± 10.2	78.0 ± 9.8	0.19
**Cholesterol (mg/dl)**	206 ± 40	186 ± 38	173 ± 29	<0.001
**Triglyceride (mg/dl)**	131 ± 52	124 ± 42	125 ± 45	0.52
**HDL-C (mg/dl)**	44 ± 9	42 ± 10	42 ± 9	0.19
**LDL-C (mg/dl)**	121 ± 25	109 ± 25	101 ± 21	<0.001


Values are means ± SD, P values obtained by ANOVA. HDL-C; High density lipoprotein-cholesterol, LDL-C; Low-density lipoprotein- cholesterol, CC; Common homozygotes, CG; Heterozygotes and GG; Rare homozygotes.

## Discussion

The atherogenic lipids have a potent activity to induce endothelium dysfunction and lesion formation (25-27). Secretory *PLA2IIa* enhances LDL oxidation and promotes the formation of bioactive phospholipids via the release of polyunsaturated free fatty acids. This can be a cause in stimulating monocyte-endothelium interaction (28). Secretory *PLA2IIa* and oxLDL are involved in inflammation and oxidative stress and are accepted as oxidative and inflammatory markers (29). Immunohistochemistry (IHC) with anti-human *PLA2IIa* demonstrated that sPLA2 is present in the peritoneal environment and is secreted by the endometrial gland (30).

The present study examined the association between the *763C>G* s*PLA2IIa* gene polymorphism in patients with endometriosis and in control subjects in Iran. We observed that the genotype distribution for this polymorphism was significantly different between the individuals with and without endometriosis. In a previous study, a strong relationship was observed between *PLA2G2A* polymorphism with serum s*PLA2IIa* levels (p<0.0001) (18). Moreover, by haplotype analysis, they reported that of the potential 64 haplotypes defined by SNPs of *PLA2G2A*, only six haplotypes occurred at a frequency >5% and these haplotypes were associated with s*PLA2IIa* levels (18).

Secretory *PLA2IIa* can modify LDL particles and it has been shown that these modified LDLs have enhanced affinity for proteoglycan and glycosaminoglycan binding (31-33). The interaction between LDL and proteoglycans, which induces LDL aggregation and fusion, is a factor that contributes to the pathogenesis. Moreover, the macrophage-specific over-expression of group IIa sPLA2 increases atherosclerosis and enhances collagen deposition (34, 35). Ploak et al. showed an increased level of oxLDL in the peritoneal fluid of women with endometriosis in advanced stages (36).

The data from our experimental study demonstrate that women with endometriosis have dyslipidemia compared with the control subjects. These findings are consistent with previous studies (11).

A study on Caucasian men and women with Type II diabetes mellitus (T2D) reported that the frequency of the minor allele at this polymorphism was 23%, 95%CI=0.20-0.25), which is similar to the frequency observed in the control group of our study (18). Furthermore, consistent with Wooton et al, which reported no effect of the *763C>G* polymorphism on HDL-C and TG levels in the Caucasian population with T2D (18), we found no association between this polymorphism and both HDL-C and TG levels in the present study. Our results showed no significant difference in HDL-C levels in individuals with genotype GG compared with those with CC genotype. As, HDL-C levels depend on different factors including diet, exercise, environment and genetics, probably, the G allele is not the sole determinant of serum HDL-C levels. Overall, we observed a significant association between *PLA2G2A* and endometriosis risk.

Our research has shown that the risk of endometriosis is lower in subjects with the G allele than individuals homozygous for the normal allele. Moreover, our findings suggest that in GG homozygote women, total cholesterol and LDL-C levels are less than those with CC genotype. Cholesterol and LDL-C, which are known inflammatory risk factors, may be involved in the occurrence and severity of disease, which may indicate beneficial effects of mutation in this gene. The *763C>G* polymorphism of *PLA2G2A* is also in linkage disequilibrium with an identified functional polymorphism in this gene that may influence the risk of endometriosis.

The main limitations of the present study were the relatively small sample size and the absence of data on sd-LDL and oxLDL particles. Another limitation of the present study was no standardization of the intensity of the physical activity of patients, a factor that may also influence the levels of serum lipids. Also, lack of data on s*PLA2IIa* activity was another limitation of this study.

## Conclusion

This study reports for the first time a significant association between *PLA2G2A* gene polymorphism with the risk of endometriosis.

Despite the *763C>G* polymorphism showing a tendency to decrease endometriosis risk, larger series are warranted to confirm this observation and to further study the association between *PLA2G2A* and endometriosis development.
